# Central Connectivity of Transient Receptor Potential Melastatin 8-Expressing Axons in the Brain Stem and Spinal Dorsal Horn

**DOI:** 10.1371/journal.pone.0094080

**Published:** 2014-04-07

**Authors:** Yun Sook Kim, Jun Hong Park, Su Jung Choi, Jin Young Bae, Dong Kuk Ahn, David D. McKemy, Yong Chul Bae

**Affiliations:** 1 Department of Anatomy and Neurobiology, School of Dentistry, Kyungpook National University, Daegu, Korea; 2 Neurobiology Section, Department of Biological Sciences, University of Southern California, Los Angeles, California, United States of America; Tokyo Medical and Dental University, Japan

## Abstract

Transient receptor potential melastatin 8 (TRPM8) ion channels mediate the detection of noxious and innocuous cold and are expressed by primary sensory neurons, but little is known about the processing of the TRPM8-mediated cold information within the trigeminal sensory nuclei (TSN) and the spinal dorsal horn (DH). To address this issue, we characterized TRPM8-positive (+) neurons in the trigeminal ganglion and investigated the distribution of TRPM8+ axons and terminals, and their synaptic organization in the TSN and in the DH using light and electron microscopic immunohistochemistry in transgenic mice expressing a genetically encoded axonal tracer in TRPM8+ neurons. TRPM8 was expressed in a fraction of small myelinated primary afferent fibers (23.7%) and unmyelinated fibers (76.3%), suggesting that TRPM8-mediated cold is conveyed via C and Aδ afferents. TRPM8+ axons were observed in all TSN, but at different densities in the dorsal and ventral areas of the rostral TSN, which dominantly receive sensory afferents from intra- and peri-oral structures and from the face, respectively. While synaptic boutons arising from Aδ and non-peptidergic C afferents usually receive many axoaxonic contacts and form complex synaptic arrangements, TRPM8+ boutons arising from afferents of the same classes of fibers showed a unique synaptic connectivity; simple synapses with one or two dendrites and sparse axoaxonic contacts. These findings suggest that TRPM8-mediated cold is conveyed via a specific subset of C and Aδ afferent neurons and is processed in a unique manner and differently in the TSN and DH.

## Introduction

Transient receptor potential melastatin 8 (TRPM8), a member of TRP channel superfamily, is a nonselective cation channel that is activated by cold temperatures and by cooling compounds, such as icilin and menthol (for review see, [1], [2], [3]). Functionally, TRPM8 is involved in the mediation of innocuous and noxious cold (∼26°C–∼8°C, [2], [4], [5]), cold-induced hypersensitivity following nerve injury and inflammation [6], and cooling-mediated analgesia [7]. Despite the large numbers of studies on TRPM8, however, little is known of where and how TRPM8-mediated cold information is transmitted and processed in the 1^st^ relay nuclei of the brainstem and in the spinal dorsal horn (DH), as well as the type and proportions of primary sensory afferents that carry it [8], [9].

Trigeminal sensory nuclei (TSN), which receive somatosensory input from the craniofacial region, are subdivided into 4 sub-nuclei based on cyto-architectonical and functional differences, the trigeminal principal (Vp), oral (Vo), interpolar (Vi), and caudal (Vc) nuclei [10]. The Vc is considered the principal relay nucleus of the brainstem for orofacial nociception (for review see, [11]) as well as thermal sensation [12]. However, recent studies, based on effects of lesioning of TSN and expression of nociceptive receptors, show that rostral TSN are also involved in the processing of orofacial nociception and thermal sensation [13], [14], [15], [16].

Central connectivity of primary afferents in the TSN and DH differs according to fiber type, suggesting that specific sensory information conveyed through each is processed in a unique manner at the 1^st^ relay nucleus. For example, boutons arising from peptidergic C fiber exhibit simple central connections with one or two dendrites and receive few axoaxonic contacts, and thus sparse presynaptic modulation [17]. However, boutons arising from non-peptidergic C fibers, Aδ fibers, and Aβ fibers typically show complex synaptic connectivity with multiple postsynaptic dendrites and frequently receive high degree of presynaptic modulation through axoaxonic contacts [18], [19], [20]. It suggests that specific peripheral sensory input conveyed via specific fiber type is processed in a distinct manner in the TSN and DH. At present, little is known about the central connectivity of TRPM8-expressing fibers in the TSN and DH, which may help understand how the TRPM8-mediated sensory information is processed. We hypothesize that cold information mediated by TRPM8, which is expressed in specific subsets of primary sensory neurons and their afferents [8], [9], may be processed in rostral TSN as well as Vc, and that the TRPM8+ axon terminals may show a unique central connectivity in the 1^st^ relay nuclei of the TSN and DH.

To test this hypothesis, we investigated TRPM8-positive (+) neurons in the trigeminal ganglion (TG), and the distribution and central connectivity of their axons and terminals in the TSN and in the DH using light and electron microscopic immunohistochemistry of serial thin sections in transgenic mice expressing a genetically encoded axonal tracer in TRPM8+ neurons.

## Materials and Methods

### Ethics statement

All animal care and experimental procedures were performed according to the National Institutes of Health guidelines for the Use of Experimental Animals to ensure minimal animal use and discomfort and were approved by the Kyungpook National University Intramural Animal Care and Use Committee (permit number: KNU 2011-44). All animals were provided food, water *ad libitum*, and housed under controlled temperature with reverse light/dark cycle conditions.

### Animals and tissue preparation

Transgenic mice expressing enhanced green fluorescent protein (GFP) by the Trpm8 transcriptional promoter were generated and their offspring were genotyped as described previously [8]. Twelve male Trpm8^GFP^ mice (weight 25–30 g), aged 6–10 weeks, were used for this study, including 9 for light microscopic (LM) immunohistochemistry (3 for immunoperoxidase and 6 for immunofluorescence staining), and 3 for electron microscopic (EM) immunohistochemistry (single immunostaining for GFP). In order to distinguish the mandibular (V3) portion of the TG from the opthalmo-maxillary (V1–V2) portion, 5% rhodamine dextran amine (RDA, 3000 MW, D3308; Invitrogen, Carlsbad, CA, USA) was injected into the lingual nerve in the tongue in the 3 mice of the 6 mice used for immunostaining (see above): The portion of the TG containing many RDA-labeled somata was defined as the V3 portion. The mice were allowed to survive for 5 days. For tissue fixation, mice anesthetized with sodium pentobarbital (80 mg/kg, i.p.) were perfused transcardially with 10 ml of heparinized normal saline, followed by 50 ml of freshly prepared fixative. For LM immunoperoxidase and immunofluorescence staining, fixative was 4% paraformaldehyde in 0.1 M phosphate buffer (PB, pH 7.4); for preembedding EM, it was a mixture of 4% paraformaldehyde and 0.01% glutaraldehyde in PB. The brainstem, DH at L4, TG and its proximal sensory roots were dissected out, postfixed in the same fixative for 2 hours at 4°C, and cryoprotected in 30% sucrose in PB. Sections were cut on a freezing microtome at 40 μm for LM or on a Vibratome at 60 μm for EM and stored in 30% sucrose in PB at 4°C.

### Light microscopic immunohistochemistry

All incubations for LM immunohistochemistry were carried out on a shaker at room temperature. For immunoperoxidase staining, sections were permeabilized with 50% ethanol for 30 minutes, treated with 3% H_2_O_2_ for 10 minutes, blocked with 10% normal donkey serum (NDS; Jackson ImmunoResearch, West Groove, PA, USA) for 30 minutes, and incubated overnight in rabbit anti-GFP antibody (1∶3,000; A11122; Invitrogen) in phosphate-buffered saline (PBS; 0.01 M, pH 7.4). After several washings with PBS and incubation with 2% NDS for 10 minutes, sections were incubated with biotinylated donkey anti-rabbit antibody (1∶200 in PBS; Jackson ImmunoResearch), rinsed in PBS, and incubated with ExtrAvidin peroxidase (1∶5,000 in PBS; Sigma, St. Louis, MO, USA) for 1 hour. Immunoperoxidase was revealed by nickel-intensified diaminobenzidine (DAB), and sections were then lightly counterstained with cresyl fast violet. Light micrographs were obtained with an Exi digital camera (Q-imaging Inc.; Surrey, CA, USA) attached to a Zeiss Axioplan 2 microscope (Carl Zeiss; Goettingen, Germany) and saved as TIFF files. The cross-sectional area of a total of 476 TRPM8+ and 1455 TRPM8-neurons with clearly visible nucleoli in the largest profile was measured in 15 sections from three TG using Image J software (NIH, Bethesda, MD, USA). Interanimal variability in cross-sectional area was not significant (one-way ANOVA), and the data could be pooled for analysis. Proportion of TRPM8+ neurons in the mandibular and opthalmo-maxillary regions of the TG were analyzed with student *t*-test.

Double immunofluorescence was performed for analysis of co-staining of Trpm8^GFP^ with calcitonin gene-related peptide (CGRP), substance P (SP), IB4, and P2X_3_: Sections of TG were permeabilized with 50% ethanol for 30 minutes, blocked with 10% NDS for 30 minutes, and incubated overnight in a mixture of rabbit anti-GFP antibody (1∶3,000; A11122; Invitrogen) and guinea pig anti-CGRP (1∶2,000; T-5027; Peninsula Labs, San Carlos, CA, USA), guinea pig anti-SP (1∶2,000; AB5892; Chemicon, Temecula, CA, USA), or guinea pig anti-P2X_3_ (1∶3,000; AB5896; Chemicon) antibodies. For immunofluorescent staining for the non-peptidergic marker *Griffonia simplicifolia* isolectin B4 (IB4), sections were preincubated with 1 μg/ml IB4 (L-1104; Vector Laboratories; Burlingame, CA, USA) for 16 hours and then reacted with rabbit anti-GFP and goat anti-IB4 (1∶3,000; AS2104; Vector Laboratories) antibodies overnight. After several rinses with PBS and incubation with 2% NDS for 10 minutes, sections were incubated in a mixture of secondary antibodies (fluorescein isothiocyanate- or Cy3-conjugated antibodies raised in donkey, 1∶200 in PBS; Jackson ImmunoResearch). Finally, sections were rinsed, mounted on slides, coverslipped with Vectashield (Vector Laboratories), and examined on a confocal microscope (LSM 510 META; Carl Zeiss). Confocal images were acquired at a same optical slice thickness for all channels, saved in TIFF format, and adjusted for contrast and brightness using Adobe Photoshop CS3 (Adobe Systems; San Jose, CA, USA).

### Electron Microscopic immunohistochemistry

For preembedding EM, cryoprotected sections of brainstem, lumbar spinal cord at L4, and the proximal sensory root of the TG were frozen on dry ice for 20 minutes and thawed in PBS to enhance penetration. Sections were pretreated with 1% sodium borohydride for 30 minutes, to remove glutaraldehyde, blocked with 3% H_2_O_2_ for 10 minutes, to suppress endogenous peroxidase, and with 10% NDS for 30 minutes, to quench secondary antibody binding sites. Sections were further incubated overnight in rabbit anti-GFP antibody (1∶1,000; A11122; Invitrogen) at 4°C. The next day, sections were rinsed in PBS for 15 minutes and incubated for 2 hours in biotinylated donkey anti-rabbit antibody (1∶200; Jackson ImmunoResearch). After a brief rinse in PBS, sections were incubated with ExtrAvidin peroxidase (1∶5,000; Sigma) for 1 hour, and the immunoperoxidase was visualized by nickel-intensified DAB. Sections were further rinsed in PB, osmicated in 1% osmium tetroxide in PB for 1 hour, dehydrated in graded alcohols, flat-embedded in Durcupan ACM (Fluka; Buchs, Switzerland) between strips of Aclar plastic film (EMS), and cured for 48 hours at 60°C. Chips containing dense staining for Trpm8^GFP^ in the TSN, DH, and proximal sensory root of the TG, were cut out of the wafers and glued onto blank resin blocks with cyanoacrylate. Serially-cut thin sections were collected on formvar-coated single slot nickel grids and stained with uranyl acetate and lead citrate. Grids were examined on a Hitachi H 7500 electron microscope (Hitachi; Tokyo, Japan) at 80 kV accelerating voltage. Images were captured from every other section through TRPM8+ boutons in the TSN and DH, and from sections containing TRPM8+ axons in the proximal sensory root of the TG with a Digital Montage software driving a cooled CCD camera (SC1000W; Gatan, Pleasanton, CA, USA) attached to the microscope, and saved as TIFF files. Serial sections of TRPM8+ boutons were analyzed for their central connectivity. The frequency of occurrence of different type of contacts per TRPM8+ bouton in each TSN and DH was analyzed with one way ANOVA and mean values were compared using Scheffe's F-test; significance was set at p<0.05. The cross-sectional area of 329 TRPM8+ axons in 9 sections of the proximal sensory root of 3 TG was measured in micrographs at ×6,000 or ×25,000 original magnification using Image J software. Interanimal variability in frequency of occurrence of different type of contact per TRPM8+ bouton, and proportion and size distribution of cross-sectional area of TRPM8+ axons within the same group was insignificant (one-way ANOVA), and the data could be pooled per group for the analysis.

### Antibodies and immunohistochemical controls

To control for specificity of primary antibodies, we processed tissues according to the above protocols, except that blocking peptides were added at various concentrations. On EM, specificity of the immunoreaction was also confirmed by the consistency of immunostaining in adjacent serial thin sections of the same axons and boutons.

The antibodies against CGRP, SP, IB4, and P2X_3_ were extensively characterized in previous studies [15], [16]. The anti-CGRP cross-reacts with human and rat CGRP, but does not cross-react with amylin and calcitonin as determined by radioimmunoassay (manufacturer's technical information). The pattern of CGRP staining in the mouse TG was similar to that in previous studies [8], [15], [21]. Specific immunostaining with CGRP antibody was abolished by preadsorption with a blocking peptide (H-1470.0500, Lot 1018258; Bachem; San Carlos, CA, USA) at a concentration of 10 μg/ml. The pattern of SP staining in the mouse TG was similar to that in previous studies [16], [22] and its specific staining was abolished by preadsorption with SP peptide (S6883, Lot 067K5110; Sigma) at a concentration of 10 μg/ml. Sections incubated with the anti-IB4 without prior incubation with IB4 showed no specific staining and the pattern of IB4 staining in the mouse TG was similar to that in previous studies [16], [23]. The specific immunostaining for P2X_3_ was completely eliminated by preadsorption with the blocking peptide (P10108, Lot P400124; Neuromics, Edina, MN, USA) at a concentration of 2 μg/ml. The staining pattern of P2X_3_ in the mouse TG was consistent with previously performed experiment [15], [24]. The rabbit anti-GFP antibody (A11122; Invitrogen) is raised against GFP protein extracted from *Aequorea victoria* and purified by ion-exchange affinity column to remove nonspecific immunoglobulin. Its specificity was confirmed in a non-GFP-expressing mouse line [25].

## Results

### Characterization of TRPM8-expressing neurons and their afferents

**Figure 1:**
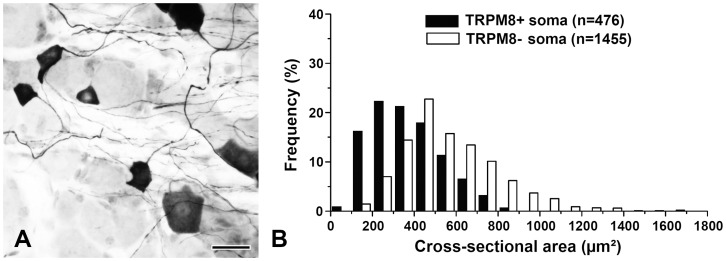
Light micrograph showing TRPM8+ neurons in the trigeminal ganglion and their size distribution. (A, B) The TRPM8+ neurons are mostly small and medium sized. Scale bar  = 25 μm.

**Figure 2:**
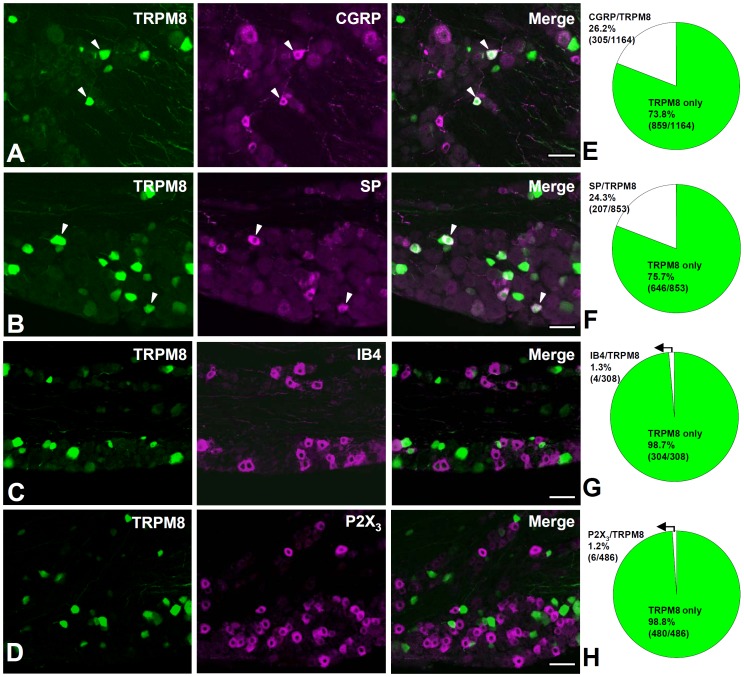
Histochemical characterization of TRPM8+ neurons in the trigeminal ganglion. (A–D) Double-immunofluorescence staining for Trpm8^GFP^ and markers for peptidergic C nociceptive neurons, CGRP (A) and SP (B), and for non-peptidergic C nociceptive neurons, IB4 (C), and P2X_3_ (D). colocalization is represented in white in the merged images (arrowheads). (E–H) Quantitative analysis of the co-staining of Trpm8^GFP^ with CGRP (E), SP (F), IB4 (G), and P2X_3_ (H). Of all TRPM8+ neurons, 26.2% (305/1164 in 12 sections) co-stain for CGRP, 24.3% (207/853 in 11 sections) co-stain for SP, 1.3% co-stain for IB4 (4/308 in 10 sections), and 1.2% co-stain for P2X_3_ (6/486 in 10 sections). Scale bars  = 50 μm.

First, we characterized TRPM8+ somata and axons in the TG. Immunoreactivity for the reporter GFP in the TG, revealed by either immunoperoxidase or immunofluorescence, was confined to the cytoplasm of the neurons. Size measurements performed in 15 sections from three mice, revealed that TRPM8+ neurons are mostly small- and medium-sized (mean cross-sectional area±S.D., 368.0±164.6 μm^2^, range, 74.5–884.6 μm^2^, n = 476, Fig. 1). Double immunofluorescence staining with other nociceptive markers showed that 26.2% of all TRPM8+ neurons costained with CGRP, 24.3% costained with SP, 1.3% costained with IB4, and 1.2%, costained with P2X_3_ (Fig. 2). The proportion of neurons labeled for TRPM8 was significantly higher in the mandibular region than in the opthalmo-maxillary region (22.1±4.2% vs. 14.2±2.7%, p<0.05), which is consistent with the previous *in situ* hybridization study in the rat [26] and suggests distinct innervation patterns between these two regions in the TG.

**Figure 3:**
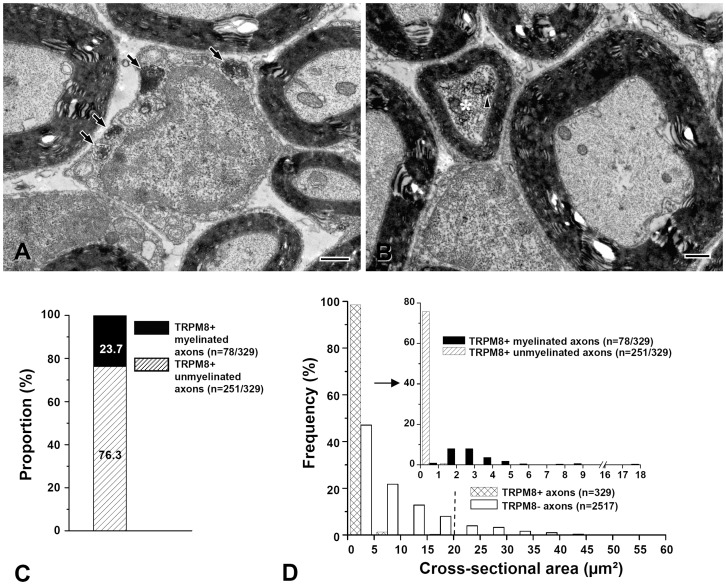
TRPM8-expressing axons in the proximal sensory root of the trigeminal ganglion. (A, B) Electron micrographs showing TRPM8+ unmyelinated (A, arrows) and small myelinated axons (B, asterisk). The immunoreaction product is indicated by arrowhead. (C, D) Stacked histograms showing the proportion (C) and size distribution (D) of the TRPM8+ unmyelinated and myelinated axons: ∼76% of TRPM8+ axons are unmyelinated and ∼24% are myelinated. All the TRPM8+ myelinated axons are small myelinated axons within Aδ fiber size range (<20 μm^2^ in cross-sectional area, left side of the dotted line). TRPM8+ axons that are larger than 20 μm^2^ in cross-sectional area (equivalent to 5 μm in diameter, right side of the dotted line in D, presumed large myelinated Aβ fibers) are not observed. Scale bars  = 500 nm.

Next we performed electron microscopic analysis of the fiber types conveying the TRPM8-mediated cold signals in the sensory root proximal to the TG, finding that large majority of the TRPM8+ axons were unmyelinated (76.3%, 0.03–0.57 μm^2^ in cross-sectional area) and the remaining 23.7% comprised by small myelinated axons in the Aδ fiber size range (<20 μm^2^ in cross-sectional area, which is equivalent to <5 μm in diameter). Conversely, immunoreactivity for GFP was not observed in large myelinated axons in the Aβ fiber size range (>20 μm^2^, Fig. 3), results consistent with TRPM8 expression in putative thermosensory and nociceptive afferent fibers.

### TRPM8-positive axons have different densities in areas of the rostral trigeminal sensory nuclei dominated by intra- and peri-oral input vs. facial input

**Figure 4:**
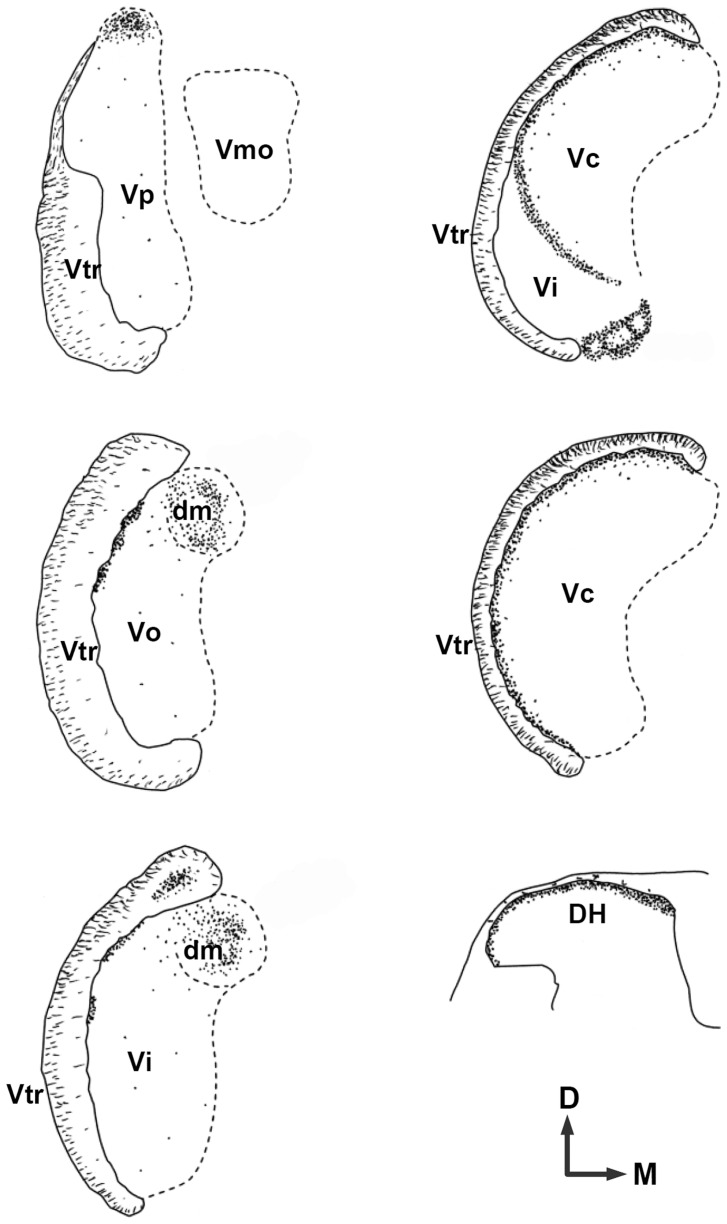
Schematic drawing showing the distribution of TRPM8+ axons and terminals in the trigeminal sensory nuclei and spinal dorsal horn (DH). TRPM8+ axons and terminals are sparse in the ventral and middle parts of the trigeminal principal (Vp), oral (Vo), and interpolar (Vi) nuclei, but dense in the lamina I and outer part of the lamina II of the trigeminal caudal nucleus (Vc), DH, and in the dorsomedial parts of Vp, Vo, and Vi, along the lateral margins bordering the trigeminal tract (Vtr) in the Vo and Vi, in the paratrigeminal nucleus, and in the caudal ventrolateral medulla. dm: Dorsomedial nucleus of Vo and Vi, D: dorsal, M: medial.

**Figure 5:**
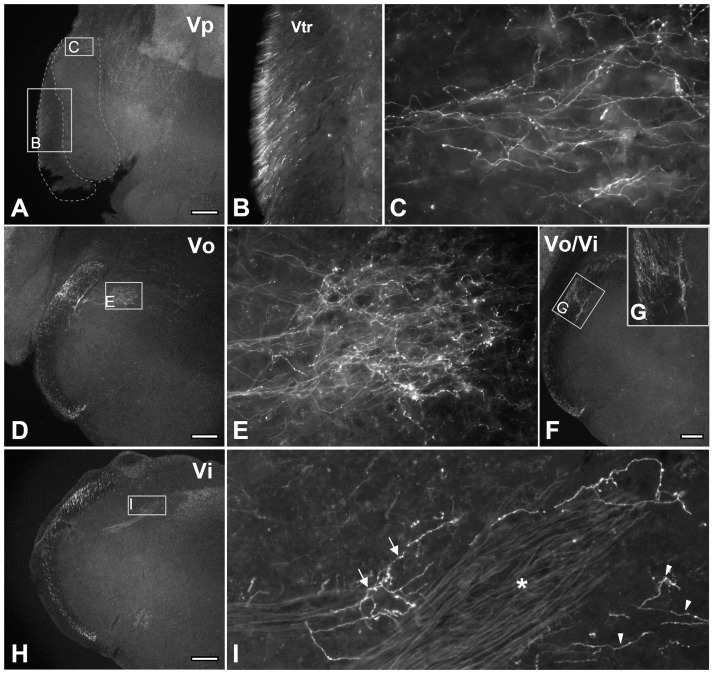
Immunofluorescence staining for Trpm8^GFP^ in axons and terminals in the rostral trigeminal sensory nuclei. TRPM8+ axons and terminals are dense in the dorsomedial parts of the trigeminal principal (Vp: A, C), oral (Vo: D, E), and interpolar (Vi: H, I) nuclei. Note the dense TRPM8+ axons in the ascending trigeminal tract (A, B), dorsal part of the spinal trigeminal tract (D, H) and in the dorsolateral margin of Vo bordering spinal trigeminal tract (F, G). In I, the portion lateral to the fiber bundles of solitary tract (asterisk) is dorsomedial part of the Vo and the portion medial to them is solitary tract nucleus: Arrows and arrowheads indicate TRPM8+ axons in the dorsomedial part of the Vo and solitary tract nucleus, respectively. B, C, E, G and I are higher magnification of boxed areas in the A, A, D, F, and H, respectively. Vtr: trigeminal tract. Scale bars  = 200 μm.

**Figure 6:**
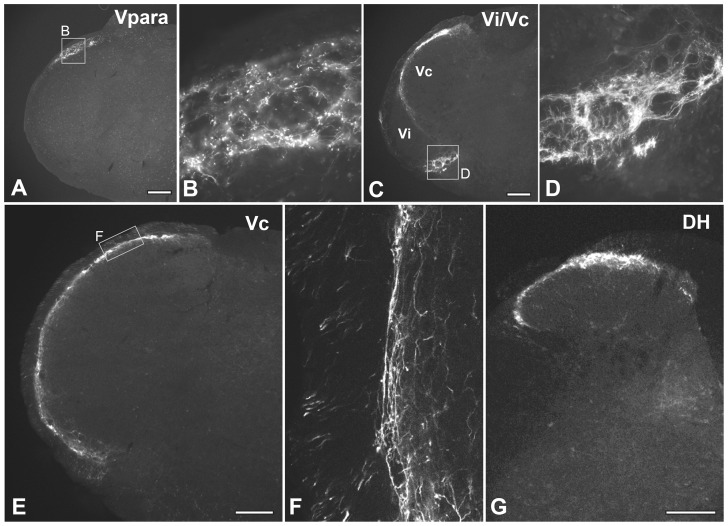
Immunofluorescence staining for Trpm8^GFP^ in axons and terminals in the paratrigeminal nucleus (Vpara), at the transition (Vi/Vc) between the trigeminal interpolar (Vi) and caudal (Vc) nuclei, in the mid-level of Vc, and the spinal dorsal horn at L4 (DH). TRPM8+ axons and terminals were dense in the Vpara (boxed area, A, B), in the caudal ventrolateral medulla (boxed area) at the Vi/Vc transition (C, D), and lamina I and outer part of lamina II of the Vc (E, F) and DH (G). B, D and F are higher magnification of boxed areas in A, C and E, respectively. Scale bars  = 200 μm.

To determine the termination pattern of putative cold-sensitive afferents and where the orofacial and somatic TRPM8-mediated cold information is relayed, we examined the distribution of TRPM8+ axons and terminals in the brainstem and DH. In the brainstem, TRPM8+ axons were found in both the ascending and descending spinal trigeminal tracts where they were dense in the external part of the tract and issued terminal axons to all subdivisions of the TSN (Fig. 4). In the rostral TSN (Vp, Vo, and Vi), TRPM8+ axons were sparse in the ventral and middle region (which predominantly receives sensory input from the face), but dense in the dorsomedial region (which predominantly receives sensory input from intra- and peri-oral areas; Figs. 4, 5), Thus, in Vp, TRPM8+ afferents issued a large number of axon collaterals and terminals into its dorsomedial part (Figs. 4, 5A–C). In Vo and Vi, these fibers were also dense in the dorsomedial parts (Vo.dm and Vi.dm; Fig. 5D–I). These findings suggest that TRPM8-mediated noxious and innocuous cold information from intra- and peri-oral areas is typically processed in the rostral TSN, while that from face is rarely processed in this region. TRPM8 innervation was also denser in the dorsal part of the spinal trigeminal tract than in the middle and ventral parts (Fig. 5D–G). Dense TRPM8+ axons and terminals were also observed in the paratrigeminal nucleus (Vpara) at the Vi level (Fig. 6A, B) and in the caudal ventrolateral medulla of the Vi/Vc transition zone (Fig. 6C, D). Caudal to this level, TRPM8+ axons and terminals were dense in lamina I and the outer part of the lamina II (IIo) throughout the dorsoventral aspect of Vc (Fig. 6E, F). In the DH, TRPM8+ axons and terminals were also dense in lamina I and IIo (Fig. 6G).

### TRPM8-positive boutons exhibit distinctive central connectivity in the brainstem and spinal dorsal horn

**Figure 7:**
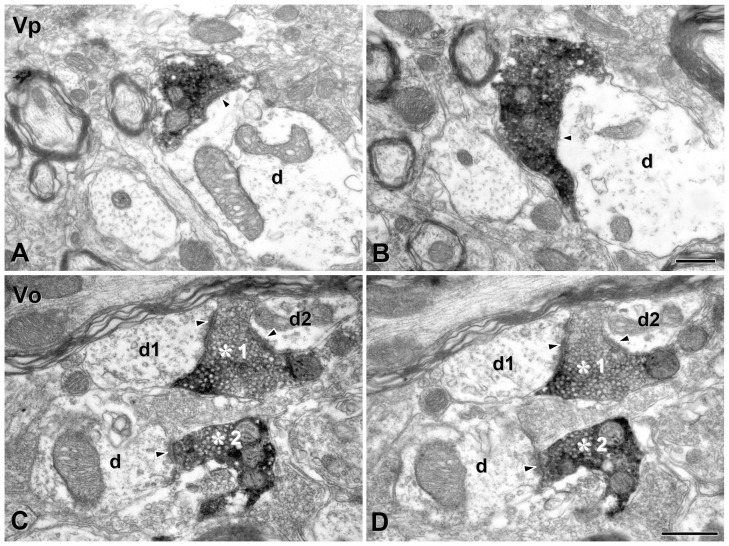
Electron micrographs of serial thin sections of TRPM8+ boutons in the dorsomedial part of the trigeminal principal (Vp) and oral (Vo) nuclei. (A, B) A TRPM8+ bouton in adjacent thin sections makes asymmetric synaptic contact with a medium-sized dendrite (d) in the Vp. (C, D) Two TRPM8+ boutons (asterisks 1 and 2) in adjacent thin sections are filled with many round vesicles in the Vo. One (asterisk 1) makes synaptic contact with two dendrites (d1 and d2) and the other (asterisk 2) with a single dendrite (d). Immunoreactivity for Trpm8^GFP^ is identifiable as electron-dense reaction product. Arrowhead indicates the site of synaptic contact. Scale bars  = 500 nm in B and D apply to A and C, respectively.

**Figure 8:**
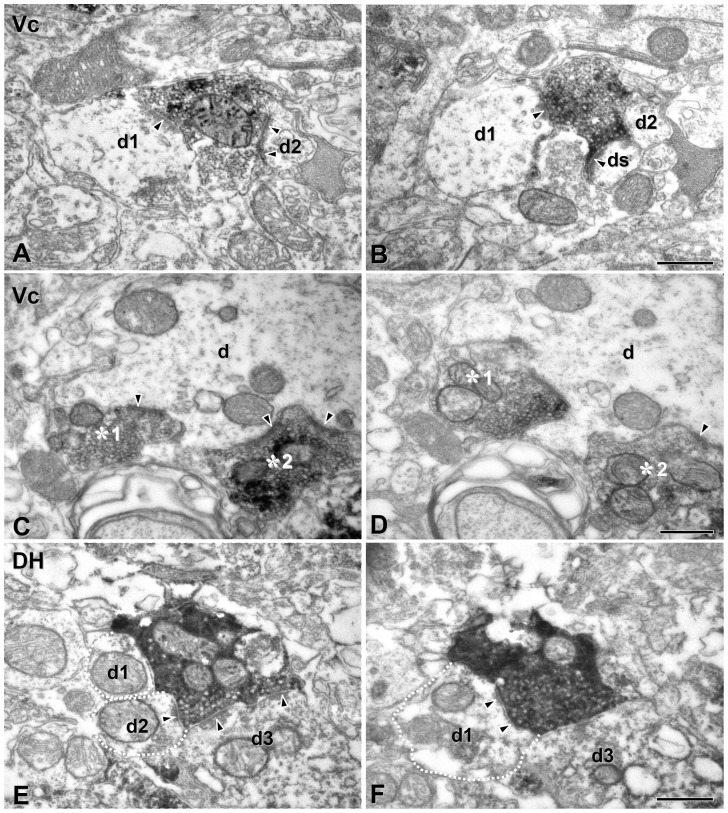
Electron micrographs of serial thin sections of TRPM8+ boutons in the superficial lamina of the trigeminal caudal nucleus (Vc) and of the spinal dorsal horn (DH) at L4 level. (A, B) A TRPM8+ bouton in adjacent thin sections makes asymmetric synaptic contacts with 3 postsynaptic profiles in the Vc: two small-sized dendrites (d1, d2), and a dendritic spine (ds). (C, D) Two TRPM8+ boutons (asterisks 1, 2) in adjacent thin sections make synaptic contacts with the same postsynaptic dendrite (d) in the Vc. (E, F) A TRPM8+ bouton in adjacent thin sections makes synaptic contacts with 3 dendrites (d1–d3). Dotted line demarcates the border of postsynaptic dendrites, d1 and d2 in the DH. Immunoreactivity for TRPM8 is identifiable as electron-dense reaction product. Arrowhead indicates the site of synaptic contact. Scale bars  = 500 nm in B, D and F also apply to A, C and E, respectively.

At the EM level, TRPM8+ axons and terminals were identified by the presence of an electron-dense reaction product in their axoplasm with the majority of the labeled boutons sinusoid or elongated in shape. These terminals were filled with numerous clear round vesicles of uniform size and occasionally contained large dense cored vesicles. We further analyzed synaptic connectivities of these boutons using reconstruction from serial sections: Most TRPM8+ boutons made asymmetric synaptic contacts with small and medium caliber dendrites or spines (Figs. 7, 8), suggesting that TRPM8-mediated cold input is transmitted to the distal and middle segment of the dendritic tree of the postsynaptic neuron, whereas synaptic contacts with somata or primary dendrites were very rare (Table 1). In Vp and Vo, virtually all TRPM8+ boutons made synaptic contacts with one or two dendrites (100% in Vp, 97.6% in Vo: Fig. 7, Table 2), yet in comparison those that made contacts with three or more dendrites were rare (0% in Vp, 2.4% in Vo). Conversely, in Vc and L4, a large number of TRPM8+ boutons made synaptic contacts with 3–5 dendrites (23.9% in Vc, 27.7% in L4; Fig. 8, Table 2). Thus, the number of postsynaptic targets and the degree of synaptic divergence at the single bouton level was significantly higher in the Vc or L4 compared to the Vp or Vo (Table 1). The number of TRPM8+ boutons receiving axoaxonic contacts from axonal endings, implying that they are subject of presynaptic modulation, were rare in all TSN and in L4 (Tables 1, 2). The pattern of central connections of TRPM8+ boutons in L4 was similar to that in Vc (Tables 1, 2).

**Table 1:** Frequency of occurrence of different types of contacts per TRPM8+ bouton in the trigeminal sensory nuclei and spinal dorsal horn^1^.

		Synaptic contacts with			Total no. of
Nucleus	No. of boutons analyzed	Soma or primary dendrite	Dendritic shaft or spine^2^	Presynaptic axonal endings	Postsynaptic profiles^2^
Vp	51	0.00±0.00 (n = 0)	1.12±0.33 (n = 57)	0.06±0.24 (n = 3)	1.12±0.33 (n = 57)
Vo	41	0.02±0.16 (n = 1)	1.12±0.46 (n = 46)	0.00±0.00 (n = 0)	1.15±0.42 (n = 47)
Vc	46	0.07±0.25 (n = 3)	1.85±1.09 (n = 85)	0.02±0.15 (n = 1)	1.91±1.07 (n = 88)
L4	47	0.00±0.00 (n = 0)	1.91±1.19 (n = 90)	0.02±0.15 (n = 1)	1.91±1.19 (n = 90)

1 Values are Mean±SD.

2 Indicates that Vp is significantly different from Vc and L4 as well as Vo (one way ANOVA, p<0.05).

n in parenthesis indicates the number of synaptic contacts with total TRPM8+ boutons in each nucleus.

**Table 2:** Frequency of occurrence (%) of TRPM8+ bouton according to the number of postsynaptic dendrites and showing axoaxonic synapse.

		No. of postsynaptic dendrites			
Nucleus	No. of boutons analyzed	1∼2	3∼4	5	Axoaxonic synapse
Vp	51	100 (n = 51)	0 (n = 0)	0 (n = 0)	5.9 (n = 3)
Vo	41	97.6 (n = 40)	2.4 (n = 1)	0 (n = 0)	0 (n = 0)
Vc	46	76.1 (n = 35)	21.7 (n = 10)	2.2 (n = 1)	2.2 (n = 1)
L4	47	72.3 (n = 34)	23.4 (n = 11)	4.3 (n = 2)	2.1 (n = 1)

n in parenthesis indicates the number of TRPM8+ boutons.

## Discussion

The main findings of the present study are that 1) TRPM8 is expressed in unmyelinated and small myelinated fibers, suggesting that TRPM8-mediated cold information is conveyed via C and Aδ fibers, 2) TRPM8+ axons project at different densities to regions of the rostral TSN, which receive predominantly intraoral input vs. facial input, suggesting that TSN involved in the processing of TRPM8-mediated cold from each intraoral and facial area are different, and 3) TRPM8+ boutons have distinctive central connectivity, suggesting that the TRPM8-mediated cold information is processed in a unique manner in each nucleus of the TSN and in the DH.

### Characterization of TRPM8-expressing primary afferent neurons

In the TG, 26% and 24% of the TRPM8+ somata coexpressed CGRP and SP, respectively, similar to what was reported previously [8]. Since 93% of the SP+ somata in the TG coexpress CGRP [27], it can be estimated that about 28% of the TRPM8+ neurons are CGRP+ and/or SP+. In addition, a small fraction of TRPM8+ neurons were IB4+ (1.3%) or P2X_3_+ (1.2%). Given that 76% of TRPM8+ axons are unmyelinated, these findings suggest that about 46.7% (76–28–1.3%) of TRPM8+ afferent neurons can be a specific subset of C afferent neurons, different from the “classical” peptidergic and non-peptidergic “C” nociceptive neurons. In the present study, TRPM8 was expressed by unmyelinated fibers (76.3%) and small myelinated fibers (23.7%), but not by large myelinated fibers (Aβ fibers), which provides a morphological evidence supporting previous electrophysiological studies showing that C and Aδ fibers are activated by noxious cold [28], [29] and innocuous cool stimuli [30], [31], [32] and that TRPM8-null mice are largely deficient in cold-evoked discharges in C and Aδ fibers [4]. The fiber pupulations expressing TRPM8 are also at variance with those expressing nociceptive receptors such as TRPV1, P2X_3_, which are vitually limited to unmyelinated C fiber, possibly reflecting their functional differences [15], [33].

Recent studies indicated that TRPM8 is expressed in two distinct populations of cold-sensitive somatosensory neurons: one with a low-activation threshold near 30°C and sensitive to menthol but not capsaicin, the other with a high-activation threshold below 20°C, sensitive to menthol, capsaicin, and ATP, and properties of a nociceptive neuron [34], [35]. The former is suggested to be the traditional cold receptor activated by innocuous cooling, the latter is likely to be the cold-sensitive nociceptor that also expresses other nociceptive markers. In the present study, the TRPM8+ only neurons may be traditional cold receptors that respond to innocuous cooling and the TRPM8+ neurons that coexpress CGRP/SP may be cold-sensitive nociceptors. Areas of the TSN where TRPM8+ afferents densely project can be classified into 2 parts according to the existence of CGRP+ terminals and response to noxious stimulation. One is the superficial lamina of the Vc and Vodm where dense CGRP+ terminals are observed and c-Fos response is evoked by the noxious stimulation of trigeminal receptive field. The other is dorsomedial part of the Vp and Vi which contain neither CGRP+ terminals [36] nor respond to noxious stimulation by c-Fos expression [37], [38], [39]. These findings can provide a notion that the superficial lamina of the Vc and Vodm, among the TSN that receive dense TRPM8+ afferents, may be mainly implicated in the cold nociception and the dorsomedial area of the Vp and Vi may be mainly implicated in the innocuous cooling.

### Projections of TRPM8+ axons to the trigeminal sensory nuclei

We speculate that the TRPM8+ axons and terminals in the TSN and DH are the anatomical substrate for TRPM8-mediated cold input from the periphery to the 1^st^ relay station in the brain stem and spinal cord. They were dense in lamina I and IIo of the Vc and DH, confirming previous observations in the DH [8], [9]. However, they were also dense in the dorsomedial part of the Vp, Vo, and Vi, suggesting that TRPM8-mediated cold information is also processed in the rostral TSN. The TRPM8+ axons and terminals were distributed differently between in the Vc and the rostral TSN: they were evenly dense throughout the dorsoventral extent of Vc, whereas in the rostral TSN, they were dense in the dorsomedial part, which predominantly receives input from the intra- and peri-oral area, and sparse in the middle and ventral parts, which predominantly receive input from the face [40]. This suggests that while the TRPM8-mediated cold information from the face is mainly processed in the Vc, that from the intra- and peri-oral area is also processed in the rostral TSN besides the Vc. The distribution of TRPM8+ axons and terminals in the dorsomedial part of the rostral TSN was similar to that of the TRPA1+ axons and terminals [16] but differed from that of the TRPV1+ axons and terminals, which are dense in the Vc but sparse in the dorsomedial part of the rostral TSN [21], suggesting that the brainstem region that is primarily involved in the processing of intra- and peri-oral cold is different from that for intra- and peri-oral noxious heat.

### TRPM8+ boutons exhibit simple synaptic connectivity

Previous studies using identification of pre- and post-synaptic neurons by intracellular and/or intra-axonal recording showed that a single axon terminal makes synaptic contact with a single dendrite of a postsynaptic neuron, suggesting that each postsynaptic dendrite arises from a different postsynaptic neuron [41], [42]. In the present study, the number of postsynaptic dendrites per TRPM8+ bouton was smaller in the Vp and Vo than in the Vc. Thus in Vp and Vo virtually all boutons showed simple synaptic connections with 1 or 2 postsynaptic dendrites, suggesting that TRPM8-mediated cold is conveyed to a specific group of postsynaptic neurons with a small degree of synaptic divergence. On the other hand, in the Vc, a considerable fraction of boutons (∼24%) made complex synaptic contacts with 3–5 dendrites, suggesting that signal from a bouton, in a considerable fraction of TRPM8+ boutons may activate multiple postsynaptic neurons that project to various regions of the CNS, forming a diffuse and divergent afferent system. While most neurons in the dorsomedial part of Vp project to the ventral posteromedial nucleus of the thalamus [43], many neurons in the dorsomedial part of Vo project to motor nuclei, including the trigeminal motor nucleus, and few project to the thalamus [42], [44]. Given this projection pattern, most TRPM8+ boutons in the Vp and Vo may be primarily involved in the discriminative perception of TRPM8-mediated cold stimuli, and in the motor response to those stimuli, respectively. Vc contains, aside from a variety of intrinsic neurons, neurons projecting to the thalamus, hypothalamus, and the parabrachial nucleus [45], [46], [47]. Since the majority of TRPM8+ boutons contact multiple postsynaptic dendrites in this nucleus, it may be simultaneously involved in the autonomic and affective responses to TRPM8-mediated cold, as well as in its sensory perception.

### TRPM8+ boutons rarely receive axoaxonic contacts

Axoaxonic synapses onto primary afferent terminals represent the morphological substrate for the presynaptic inhibition [48], [49], a mechanism for sharpening of the sensory perception such as spatial resolution. Primary afferent terminals differ in the frequency of the axoaxonic contacts they receive, according to the fiber types from which they arise. Thus, while peptidergic C-afferent boutons lack axoaxonic contacts [17], [50], non-peptidergic C-afferents receive many axoaxonic contacts and form complex synaptic arrangements with multiple pre- and post-synaptic elements [18]. In addition, a considerable fraction of boutons from Aβ, low threshold mechanoreceptive afferents (40–100%; [20], [51], [52], [53]) and Aδ high-threshold mechanoreceptive afferents (20–60%; [19]) receive axoaxonic synapses in the TSN and DH. Here, the axoaxonic synapses onto TRPM8+ boutons were very rare (0–6%) in the TSN and DH, suggesting that peripheral TRPM8-mediated cold inputs may be transmitted to postsynaptic neurons at the 1^st^ relay nucleus with little alteration. This pattern is that of the peptidergic C afferents but unlike that of the non-peptidergic C afferents, Aδ afferents, and low threshold mechanoreceptive Aβ afferents. This suggests that a large number of TRPM8+ boutons arising from SP(-) and/or CGRP(-) non-peptidergic C-fibers (∼46.7%) and Aδ fibers (23.7%), do not exhibit the central connectivity typical for these fiber types, but rather belong to a specific subgroup of C and Aδ fibers. That the TRPM8+ boutons show different central connectivity from other somatic channels also supports the notion that the cold channel is a unique channel and TRPM8-mediated cold is processed in a unique manner in the CNS.
